# Evaluation of stroke health education for primary school students in Dali, China

**DOI:** 10.3389/fpubh.2022.861792

**Published:** 2022-08-02

**Authors:** Yunjuan Yang, Jing Dai, Jieqing Min, Zhizhong Song, Shun Zha, Litao Chang, Jiajia Chai, Youpei Yang, Yang Liu, Xin Zhang, Xiyun Wu, Yuwen Gong, Xin Wang, Fang Li, Haiyan Qin

**Affiliations:** ^1^Department of School Health, Yunnan Provincial Center for Disease Control and Prevention, Kunming, China; ^2^Public Health School, Kunming Medical University, Kunming, China; ^3^Public Health School, Dali University, Dali, China; ^4^School of Economics and Management, Kunming University of Science and Technology, Kunming, China; ^5^Cardiac Ultrasound Department, Kunming Children's Hospital, Kunming, China; ^6^City College, Kunming University of Science and Technology, Kunming, China; ^7^Office, Dali Centers for Disease Control and Prevention, Dali, China; ^8^School of Economics and Management, Xi'an Jiaotong University, Xi'an, China; ^9^Society and People School, Renmin University of China, Peking, China; ^10^Infection Control Department, The First People's Hospital of Kunming, Kunming, China

**Keywords:** stroke, health education, effect, children, evaluation, ethnic

## Abstract

**Objectives:**

To provide us with some evidence to develop more targeted stroke intervention strategies, improve the health awareness of stroke among children, and advocate the health promotion campaign of “small hands holding big hands” among parents and children, we have conducted a health education program on stroke among primary school students in Dali.

**Methods:**

This study has applied stratified random cluster sampling in Dali of Yunnan, China. We compared the improvement of students' knowledge of stroke before and after our health education program in primary school students of Dali in October 2020. Data were collected through 3 rounds of survey by using the same questionnaire.

**Results:**

There were 215 participants aged 7–8 years old sampled in the first phase of the study and 145 participants in the follow-up study. The knowledge of stroke among the participants was relatively low in the pre-intervention survey. After the health education, all the indicators on stroke knowledge were improved. The correct rates in stroke definition, sequelae, and “1-2-0” identification were increased from 0 to 66.05%, to 53.95% and 64.19%, respectively, in both pre-intervention and post-intervention surveys. The correct rate of stroke knowledge was about 4.83–92.41% 3 months after the intervention. The mean score of the questionnaire was 4.25 ± 0.19 in the pre-intervention survey, and that was 15.85 ±0.27 in the post-intervention one. The mean score was 14.02 ± 0.28 post-3-month test. The score in the 3-month survey after the intervention was 11.55% lower than that in the post-intervention score.

**Conclusions:**

The effect of stroke-related knowledge in the health education program for children is improved significantly and this can last for 3 months but it also had attenuation. We should repeat pertinent health education among students.

## Introduction

According to the WHO, cardiovascular diseases (CVDs) are the leading cause of death globally, with an estimated 17.9 million lives taken each year. Also, the cause of over four out of five CVD deaths is attributed to heart attacks and strokes (1). Today, China bears a heavy burden of CVD (2). In 2018, the crude death rate of stroke was 149.49/100,000 among Chinese residents, accounting for 22.33% of the total number of deaths. Stroke was ranked as the third leading cause of death in China (3). A study has shown that the incidence of stroke was 6.2% among Bai Ethnic people aged over 35 years in Jianchuan County, Dali of Yunnan in 2018 (4), which was higher than that of adults in China. The rapid changes in lifestyle and dietary habits have made younger people more vulnerable to stroke in recent years (5). Children who had experienced early childhood stroke were at particular risk of alterations in cognitive functions (6). Moreover, epidemiological evidence of stroke burden among Dali residents was lacking. Only patient-specific studies have shown that the factors associated with stroke in Dali residents were age, blood glucose, blood lipids, daily barometric pressure, and daily air temperature (7, 8). Therefore, it is important to improve children's knowledge of stroke and its prevention.

Health education among primary school students has proved to be significantly effective in many studies (9–11). The students were in the critical age of forming the right health concepts with strong learning acceptance, moreover, their health literacy could affect that of the whole family (9, 12, 13). In the Netherlands, students can play a vital role when a stroke occurs by recognizing the signs of a stroke and calling 911 immediately (14). Less research was carried out and reported in China. The reported studies in foreign countries have mainly adopted FAST (Face, Arm, Speech, and Time) to capture a memory in stroke education (15). However, this did not work well due to the language barrier among Chinese adults (16). There have been no related studies on the methods of stroke health education and its effectiveness evaluation among ethnic children.

This study has selected the children as our study population and Dali prefecture as our survey location. It was the first intervention to improve children's stroke awareness in Yunnan. The study aimed to explore the methods of stroke health education for children and to improve children's awareness of stroke health. It is expected to provide us with some evidence for developing more targeted stroke intervention strategies in Yunnan.

## Materials and methods

### Study design

The study was a follow-up study with data that were generated from the Students' Surveys carried out in Dali of Yunnan from October 2020 to January 2021. The survey was conducted in Dali City of Yunnan province. According to the list of primary schools in Dali City, the survey school was randomly sampled by the end number of the random selecting banknotes. The survey grades and classes were also randomly sampled in the survey school by the end number of the random selecting banknotes. The study participants were recruited by cluster sampling from a sample primary school in Dali City, Yunnan, taking both representativeness and randomness into account. All the students in grades 1 and 2 were eligible to be recruited for our study. We provided stroke health education for the recruited students, and evaluated the educational effect before and after intervention in 2020 and that in 3 months after intervention in 2021. The surveyed students remained consistent from October 2020 to January 2021. Those who were not willing to sign the informed consent were excluded. All participants and/or their parents/guardians provided written informed consent before the survey. There were 215 participants aged 7–8 years old in the first phase of the study. Affected by the epidemic of COVID-19, the follow-up study used an online survey tool WJX.cn (http://www.wjx.cn to collect data. The contents were kept the same in different surveys of this study. In the follow-up survey, only 145 students met the deadline for the online questionnaire.

### Data collection

Data were collected through a questionnaire, which was designed by the research group and used in all the study surveys to measure the participants' understanding of stroke. The questionnaire mainly collects information about the participants' demographic features, their knowledge about stroke and its sources, such as its definition, attacking organ, risk factors, symptoms, identification (three-step method of identification of various problems), treatments, related sequelae, and meanings of about 120 “strokes”. The full score of this questionnaire was 22, with 7 on risk factors of stroke, 5 on typical symptoms, 2 on treatment methods, 4 on sequelae, and 4 on “Stroke 120”.

**Figure 1:**
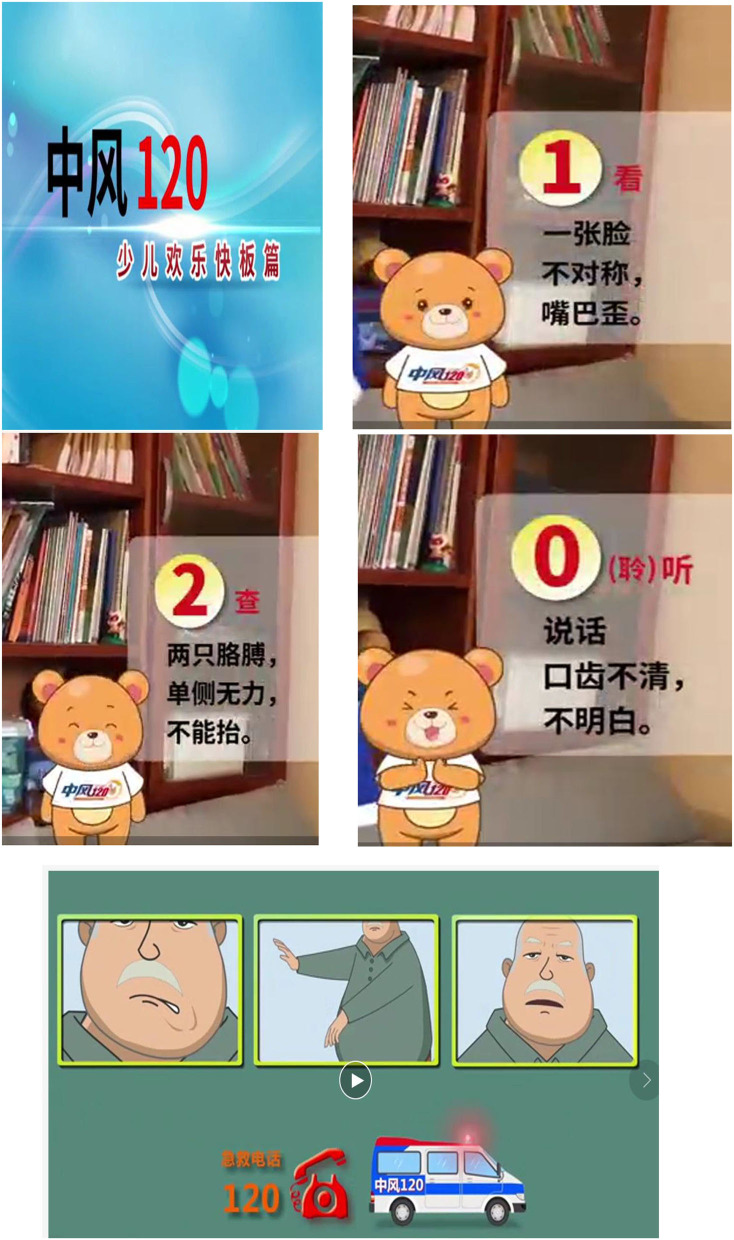
The educational videos for stroke 1-2-0.

Before the intervention, students filled out the pre-intervention questionnaire, from which we generated our baseline data. Then, participants watched a cartoon educational video, “Stroke 1-2-0” (1-2-0 indicated a three-step identification method) (Figure 1). The message delivered in this video included the following: “1” face droop, “2” arm weakness, “0” speech affected/slurred,” and “120” to call the ambulance. After watching the video, some participants shared their personal experiences about stroke, and what they would have done differently based on their new knowledge learned. After this, a professional investigator performed 1-2-0 again and introduced the meaning of stroke, its risk factors, symptoms, treatments, and related sequelae. The Q and A was finally added to enhance the students' understanding of stroke.

Assessment of the improvement in students' knowledge was carried out twice by the same questionnaires. One was carried out immediately after the education (post-intervention), and the other was 3 months after intervention (follow-up). The three-month follow-up questionnaire was given without noticing and collected data online by a web application of “questionnaire star”. The students had limited time of answering the questions. The students' completion times were studied qualitatively before and after the field intervention (pre-intervention and post-intervention).

### Statistical analyses

The primary data analysis was conducted in 2021. All data were analyzed using SPSS 21.0 software. Differences in the mean score of survey questionnaires were compared using the ANOVA test. The correct answer rates for the questionnaire in different surveys (pre-intervention, post-intervention, and follow-up after 3 months) were calculated and stratified by gender, ethnicity, and grade subgroups. Univariate methods were not used to estimate *P*-value for differences by gender, ethnicity, and grade because high statistical power was achieved from the large sample sizes. Statistical significance was defined as *P* < 0.05.

## Results

### Population demography

A total of 215 children of 7- and 8-year-old primary school students were included and intervened, and they completed both pre-intervention and post-intervention questionnaires in October 2020. All of those who had received the intervention and completed survey questionnaires were included in the analysis, with 113 boys and 102 girls. The average age was 7.59 ± 0.49 years old. There were 84 students of Han people, 92 Bai ethnic people, and 39 people of other ethnicities. No significant difference was observed in the distribution of participants between boys and girls (*P* > 0.05), and only 145 of them completed the follow-up questionnaire in January 2021.

### Baseline data in pre-intervention

As shown in Table 1, the Chi-square test found a significant difference in terms of treatments among the students whose mothers held different jobs (*x*^2^ = 6.15, *P* = 0.05) in pre-intervention. There were no differences in other indicators. The students' knowledge of stroke was relatively poor in pre-intervention, especially in its definition, sequelae, and “1-2-0” identification. No one knew them.

**Table 1:** The correct rate of stroke knowledge of primary school students in pre-intervention in Dali, Yunnan, China.

**Subsection**	**N**	**Stroke definition**	**Attacking organ**	**Risk factors**	**Symptoms**	**Treatments**	**Sequelae**	**1-2-0 identification**
		***N* (%)**	***N* (%)**	***N* (%)**	***N* (%)**	***N* (%)**	***N* (%)**	***N* (%)**
**Gender**
Boys	113	0	39 (34.51)	2 (1.77)	1 (0.88)	2 (1.77)	0	0
Girls	102	0	34 (33.33)	2 (1.96)	1 (0.98)	3 (2.94)	0	1 (0.98)
**Age**
7	88	0	25 (28.41)	0	1 (1.14)	3 (3.41)	0	0
8	127	0	48 (37.80)	2 (1.57)	3 (2.36)	2 (1.57)	0	1 (0.79)
**Ethnic**
Han	84	0	27 (32.14)	2 (2.38)	2 (2.38)	2 (2.38)	0	0
Bai	92	0	31 (33.70)	2 (2.17)	0	1 (1.09)	0	1 (1.12)
Other	39	0	15 (38.46)	0	0	2 (5.13)	0	0
**Area**
Country	89	0	31 (34.83)	2 (2.25)	1 (1.12)	3 (3.37)	0	1 (1.12)
City	126	0	42 (33.33)	2 (1.59)	1 (0.79)	2 (1.59)	0	0
**Father's ethnics**
Han	119	0	40 (33.61)	3 (2.52)	2 (1.68)	3 (2.52)	0	1 (0.84)
Bai	70	0	23 (32.86)	1 (1.43)	0	1 (1.43)	0	0
Other	26	0	10 (38.46)	0	0	1 (3.85)	0	0
**Mother's ethnics**
Han	111	0	35 (31.53)	3 (2.70)	2 (1.80)	2 (1.80)	0	0
Bai	78	0	28 (35.90)	1 (1.28)	0	1 (1.28)	0	1 (1.28)
Other	26	0	10 (38.46)	0	0	2 (7.69)	0	0
**Father's education level**
Low	15	0	6 (40.00)	0	0	0	0	0
Middle	92	0	26 (28.26)	1 (1.09)	1	3 (3.26)	0	0
High	108	0	41 (37.96)	3 (2.78)	1	2 (1.85)	0	1 (0.93)
**Mother's education level**								
Low	15	0	3 (20.00)	0	0	0	0	0
Middle	105	0	33 (31.43)	1 (0.95)	1 (0.95)	4 (3.81)	0	0
High	95	0	37 (38.95)	3 (3.16)	1 (1.05)	1 (1.05)	0	1 (1.05)
**Father's job**
Physical worker	192	0	62 (32.29)	3 (1.56)	2 (1.04)	5 (2.60)	0	0
Brain worker	23	0	11 (47.83)	1 (4.25)	0	0	0	1 (4.35)
**Mother's job**
Physical worker	184	0	57 (30.98)	4 (2.17)	2 (1.09)	5 (2.71)^*^	0	1 (0.54)
Brain worker	31	0	16 (51.61)	0	0	0	0	0

N = 215, ^*^P < 0.05.

### Assessment of the stroke knowledge levels in pre-intervention, post-intervention, and follow-up in 3-month surveys

Students took an average of 20 min to finish the questions at the beginning of the survey. Then, the time of answering the same questions took an average of 10 min in the post-intervention survey. The time of answering the questionnaire in the post-intervention survey was way shorter than that in pre-intervention. The speed of the response to the stroke questions post-intervention was quicker than that of pre-intervention.

The Chi-square test found a significant difference in the students' levels of stroke-related knowledge in pre-intervention, post-intervention, and follow-up surveys (*P* < 0.01).

**Figure 2:**
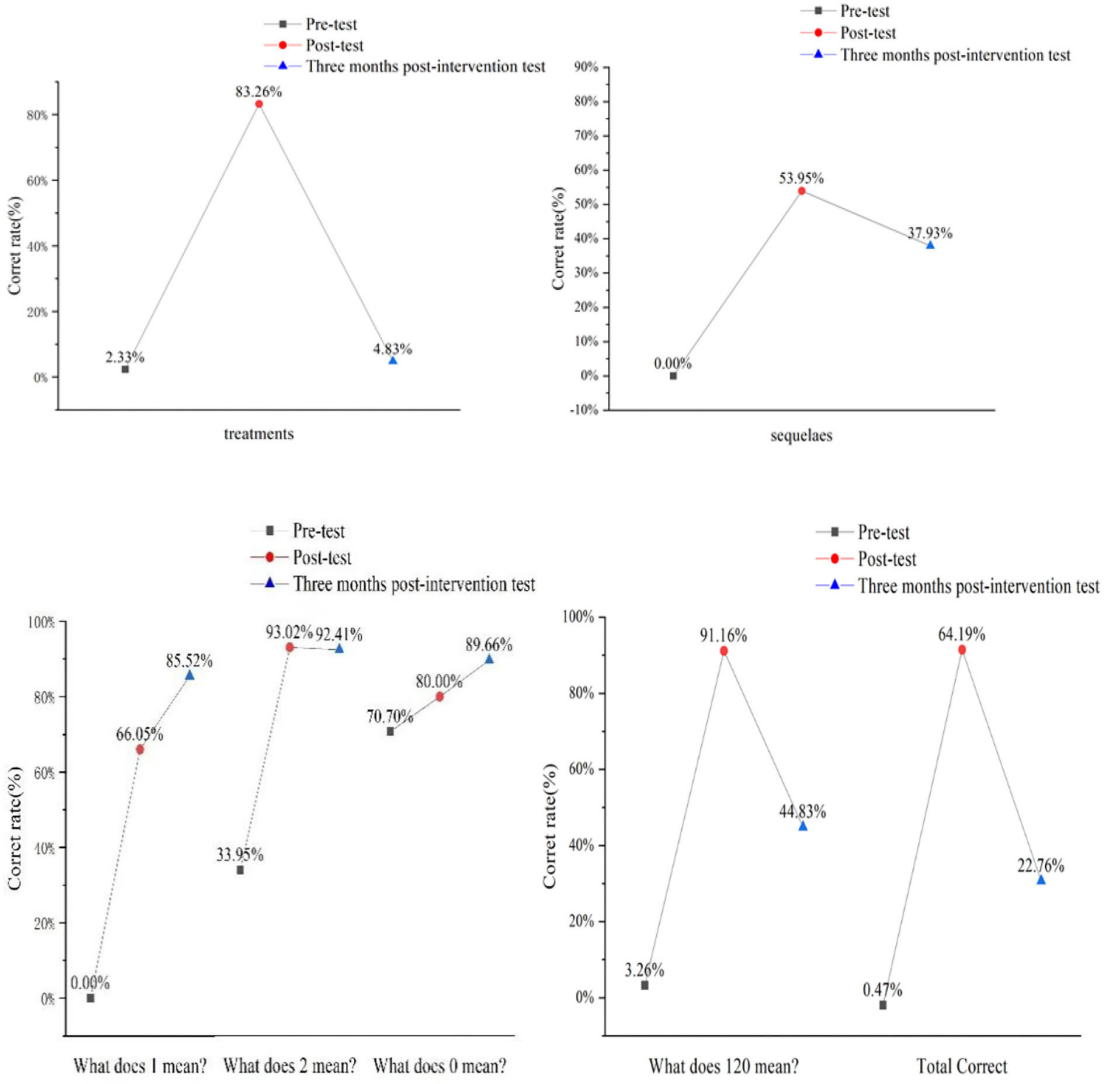
Assessment of the stroke knowledge in pre-intervention, post-intervention, and follow-up surveys.

As shown in Figure 2, after the health education, all the indicators of stroke knowledge were improved. The correct rates of the questionnaires in two post-intervention surveys were higher than that in pre-intervention, especially in the immediate post-intervention survey. However, the correct rate in the follow-up survey was lower than that in the immediate post-intervention one. The correct rate of stroke knowledge was about 4.83–92.41% (the rate is of different questions) 3 months after the intervention. The indicators that achieved better intervention effect were stroke definition, stroke organ, and the meaning of 1-2-0.

### Health education effect

As shown in Table 2, the ANOVA test found a significant difference in all indicators to measure stoke knowledge of in three surveys. The mean score of the overall questionnaire in pre-intervention was 4.25 ± 0.19., which increased to 15.85 ± 0.27 in the post-intervention. Then, it dropped to 14.02 ± 0.28 in the 3-month follow-up test. The mean score in the 3-month follow-up test was 11.55% lower than that in the post-intervention score. Despite that, the students' knowledge of stroke was improved after our intervention (*F* = 681.21, *P* < 0.01), especially in terms of stroke risk factors, symptoms, sequelae, and 1-2-0 identification. In the follow-up survey, the mean scores were also low in terms of stroke attacking organs, its treatments, and “1-2-0” identification (Table 2).

**Table 2:** The pre-intervention, post-intervention, and follow-up evaluation of stroke knowledge score of primary school students for stroke in Dali, Yunnan, China (means ± SD).

**Subsection**	**Pre-intervention**	**Post-intervention**	**Follow-up**	***F***	***P***
	**(*N =* 215)**	**(*N =* 215)**	**(*N =* 145)**		
Stroke definition	0.00 ± 0.00	0.66 ± 0.03	0.86 ± 0.03	331.82	0.00
Attacking organ	0.34 ± 0.03	0.93 ± 0.02	0.92 ± 0.02	184.18	0.00
Risk factors	2.07 ± 0.12	5.37 ± 0.14	5.36 ± 0.14	211.71	0.00
Symptoms	1.17 ± 0.08	3.91 ± 0.09	3.25 ± 0.09	307.58	0.00
Treatments	0.27 ± 0.03	1.80 ± 0.03	0.57 ± 0.05	524.17	0.00
Sequelaes	0.40 ± 0.05	3.19 ± 0.08	3.07 ± 2.91	581.21	0.00
1-2-0 identification	0.05 ± 0.02	3.29 ± 0.08	1.59 ± 0.15	428.81	0.00

### Source of health information

The top three sources of health knowledge were television (57.22%), doctors (55.83%), and teachers or school bulletin boards (55.56%).

## Discussion

This study was the first to conduct a localized health educational intervention for primary children in Dali City of Yunnan, China. It has generated some useful information to inform health education on stroke for children and provide insights into preventing and controlling children's stroke in Yunnan ethnic prefectures.

It is evident from our study results that students' knowledge has been greatly improved in terms of stroke and its identification. For example, the correct rates of stroke definition, sequelae, and “1-2-0” identification were increased from 0 to 66.05%, to 53.95 and 64.19%, respectively, after the intervention. In addition, the correct rate of stroke definition kept increasing to 85.52% in 3 months after the intervention. However, the improvement was not observed 3 months after the intervention. The correct rates of sequelae and “1-2-0” identification were decreased to 37.93 and 22.76%, respectively, in 3 months after the intervention. The mean score in the follow-up survey was 11.55% lower than that in the post-intervention one. This decrease could be explained by Ebbinghaus' memory curve. This indicated that the health education program on stoke had attenuation despite the effects achieved among children. The correct rate of stroke knowledge was about 4.83–92.41% in 3 months after the intervention, which was lower than that in the study of Shigehatake (56%) (16) and Li X in Shanghai (84.4%) (17). Therefore, we should repeat pertinent health education programs among students.

The materials used in this health education program was presented in cartoons and hip-hop song. The study found that the manner of delivering stroke health education to children was essential to ensure the effectiveness of health education. Age-targeted education can significantly raise the awareness of stroke in children. In the following study, we will pay attention to making the health education materials match the cognitive characteristics of children in the context of local culture (18, 19).

Meanwhile, we should also note that the top three sources of health knowledge were television (57.22%), doctors (55.83%), and teachers or school bulletin boards (55.56%). This indicated that the principal role of doctors, teachers, or school bulletin boards cannot be ignored in children's health education. School health education has traditionally been an effective public health strategy to help children develop a proper health concept. A study also confirmed that school health education had always been a good option for students to receive health education (20, 21). Because chronic diseases have a long progressing trajectory, children should be the priority group for future intervention in chronic disease prevention. We should carry out child-targeted health education on disease control and prevention in schools, and facilitate children to develop a healthy lifestyle, which would further eliminate their risk behaviors as early as possible and reduce the incidence of chronic diseases in young adults.

This study found that it was important to develop child-friendly health education materials in the future. Thus, how to develop a series of educational electronic animations and pamphlets for children will be the focus of future research.

## Limitations

This study has a few limitations. Our study did not have a control group to measure the actual intervention effects, so the evaluation results had certain limitations. We will further expand the study population and scope in the future study and to provide generate robust evidence for the importance of stroke health education in children.

## Conclusion

In conclusion, this study is the first one that utilized the successive follow-up data of 7–8 school-aged children of Bai ethnicity in Dali City of Yunnan, China. Especially, this study compared the effect of health education on stroke before and after intervention, even 3 months after intervention. The study revealed our intervention had significantly improved the knowledge of stroke among Bai ethnicity of 7–8 years old children of Yunnan, China.

In view that a number of benefits of conducting health education among children, such as effectively improving the health awareness of stroke, reducing high-risk behaviors, and enhancing the healthy lifestyle of the family, we should develop a health education model that could fit in with the psychology development of children and local culture to raise children's awareness on stroke prevention and control.

## Data availability statement

The raw data supporting the conclusions of this article will be made available by the authors, without undue reservation.

## Ethics statement

The survey was approved by the Medical Research Ethics Committee of Yunnan Preventive Medical Institute. Written informed consent to participate in this study was provided by the participants' legal guardian/next of kin. Written informed consent was obtained from the individual(s), and minor(s)' legal guardian/next of kin, for the publication of any potentially identifiable images or data included in this article.

## Author contributions

YJY: conducted data collection, intervention, statistical analysis and manuscript design, writing, and editing. JD: conducted field survey, collected data, provided suggestions, and manuscript preparation. JQM: field survey, advised, conceived, and designed the work, and edited the final version of the manuscript. ZZS, SZ, and LTC: coordinated survey fields and advised. YL: analyzed data and drew figures. XW and HYQ: conducted field survey, collected data, ill-disposed data, and analyzed data. JJC: provided suggestions, assisted in an English manuscript, and edited the manuscript. FL: analyzed data and provided suggestions. YPY: coordinated survey fields and conducted this study. YJY, JD, and JQM have full access to all the data in this study and takes primary responsibility for the final content. All authors have read and approved the final version of the manuscript.

## Funding

This study was supported by the Academic Leader of Medical Science in Yunnan (Grant No. D-2018007), the 16th batch of young and middle-aged academic and technical leaders in Kunming (Grant No. KMRCD-2018011), Xishan Prefecture Science and Technology Bureau Project (Grant No. 34 Xikezi), and the National Natural Science Foundation of China (71764014).

## Conflict of interest

The authors declare that the research was conducted in the absence of any commercial or financial relationships that could be construed as a potential conflict of interest.

## Publisher's note

All claims expressed in this article are solely those of the authors and do not necessarily represent those of their affiliated organizations, or those of the publisher, the editors and the reviewers. Any product that may be evaluated in this article, or claim that may be made by its manufacturer, is not guaranteed or endorsed by the publisher.
